# Radiation Dose Aspects of Hepatic Artery Infusion Chemotherapy in Uveal Melanoma Patients with Liver Metastases

**DOI:** 10.1007/s00270-022-03130-1

**Published:** 2022-04-18

**Authors:** Sebastian Zensen, Marcel K. Opitz, Johannes M. Ludwig, Johannes Haubold, Heike Richly, Jens T. Siveke, Jens M. Theysohn, Michael Forsting, Denise Bos, Benedikt M. Schaarschmidt

**Affiliations:** 1 Institute of Diagnostic and Interventional Radiology and Neuroradiology, University Hospital Essen, Hufelandstraße 55, 45147 Essen, Germany; 2 Department of Medical Oncology, West German Cancer Center, University of Duisburg-Essen, Essen, Germany; 3 Bridge Institute of Experimental Tumor Therapy, West German Cancer Center, University Medicine Essen, Essen, Germany; 4 Division of Solid Tumor Translational Oncology, German Cancer Research Center (DKFZ) and German Cancer Consortium (DKTK), Partner Site Essen, Heidelberg, Germany

## Abstract

**Purpose:**

In uveal melanoma patients, liver metastases can be treated by hepatic artery infusion chemotherapy (HAIC). During this procedure, melphalan or, less frequently, fotemustine is infused into the hepatic artery or the hepatic lobe arteries in regularly repeated interventions to achieve local tumor control. The aim of this study was to investigate the radiation exposure of HAIC.

**Material and methods:**

In this retrospective study, dose data from 841 procedures in 140 patients (mean age 65.3 ± 9.9 years, 74 female) who underwent HAIC between 06/2017 and 10/2021 at one of three different angiography systems were analyzed.

**Results:**

In the overall population, dose area product (DAP) (median (IQR)) was 1773 cGy·cm^2^ (884–3688). DAP was significantly higher in the first intervention, where a complete diagnostic workup of the vasculature was performed, than in follow-up interventions: 5765 cGy·cm^2^ (3160–8804) versus 1502 cGy·cm^2^ (807–2712) (*p* < 0.0001). DAP also increased significantly with the number of infusion positions (median, (IQR)): one position 1301 cGy·cm^2^ (633–2717), two positions 1985 cGy·cm^2^ (1118–4074), three positions 6407 cGy·cm^2^ (2616–11590) (*p* < 0.0001).

**Conclusion:**

In uveal melanoma patients with liver metastases undergoing HAIC, radiation exposure is significantly higher both at the first intervention compared to follow-up interventions, but also with increasing number of infusion positions.

Level of evidence: 3

## Introduction

Uveal melanoma (UM) is the most common primary malignancy of the eye and accounts for approximately 5% of all melanomas [1, 2]. Despite generally aggressive local tumor therapy, approximately 50% of all patients develop metastases, which are located most frequently in the liver [3–5]. Due to diffuse metastatic spread in this organ, liver-directed therapies such as transarterial chemoembolization (TACE), radioembolization (RE), or hepatic artery infusion chemotherapy (HAIC) are the primary treatment options [6, 7]. Particularly, repeated HAIC is a well-tolerated procedure, which has been shown to prolong progression-free survival with less severe hematologic adverse events compared to intravenous chemotherapy [8]. Due to the necessity to repeat this intervention regularly, radiation exposure should be diminished for the interventional radiologist and the patient alike [9].

To our knowledge, data on radiation exposure of HAIC in patients with hepatic metastatic UM are limited [10]. This shortcoming shall be addressed by this study.

## Material and Methods

### Patient Cohort

Between June 2017 and October 2021, dose data of patients who underwent HAIC at our center were included in this retrospective study. Ethical approval was granted by the local ethics committee, and the requirement to obtain informed consent was waived (21-10256-BO).

### Angiography Systems

All procedures were performed at one of three different angiography systems: the biplane angiography systems Artis Q biplane (Siemens Healthineers, Erlangen, Germany), Allura Xper FD20/10 system (Philips Healthcare, Eindhoven, The Netherlands), or the monoplane angiography system Artis zee MP (Siemens Healthineers, Erlangen, Germany). All systems are equipped with automatically controlled dose rate systems. All examinations were performed in monoplanar mode. Characteristic tube voltage was 70 kV. During all examinations, pulsed fluoroscopy was used. The pulse rate was chosen at the discretion of the interventional radiologist.

### Hepatic Artery Infusion Chemotherapy Procedure

Standard HAIC was performed as described by Heusner et al. via a transfemoral access [7]. Coil embolization for flow distribution or vessels supplying extrahepatic organs, such as a prominent right gastric artery, was performed to avoid extrahepatic spread of the chemotherapeutic agent. To ensure a homogenous distribution of the chemotherapeutic agent, one (proper hepatic artery), two (mostly right and left hepatic artery), or three infusion positions (mostly right hepatic artery, lateral and medial left hepatic artery) were used. For follow-up interventions, the previous infusion positions were used. The chemotherapeutic agent was dissolved in 50 ml and applied by an automated injector, under intermittent position control by fluoroscopy. All patients started with 40 mg of melphalan, which was increased to a maximum of 50 mg, or the chemotherapeutic agent was switched to fotemustine in case of progression. Usually, HAIC was repeated every 6–8 weeks in our department.

### Dose assessment

Dose measurements were extracted from the Digital Imaging and Communications in Medicine (DICOM) header and from the Radiation Dose Structured Report stored in the Picture Archiving and Communication System (PACS). Radiation exposure was determined in terms of dose area product (DAP).

### Statistics and Data Analysis

Statistical analysis was performed using GraphPad Prism 5.01 (GraphPad Software, San Diego, USA). To determine normal distribution, Kolmogorov–Smirnov, Shapiro–Wilk, and D’Agostino–Pearson test was applied. Normally distributed data are reported as mean ± standard deviation (SD), non-normally distributed data as median and interquartile range (IQR). Mann–Whitney U test was used for comparison of DAP between first HAIC and follow-up interventions and between HAIC with and without coil embolization. Kruskal–Wallis test with Dunn–Bonferroni post hoc test was performed for the comparison of DAP of HAIC as a function of the number of infusion positions. A *p*-value ≤ 0.05 was considered statistically significant.

## Results

### Patient Cohort

In our retrospective study, 841 HAICs performed between June 2017 and October 2021 in 140 patients could be included for evaluation. Mean age at first HAIC was 65.3 ± 9.9 years (range 39–85 years). 52.9% (74/140) of patients were female. The median number of HAICs per patient during the study period was four interventions (IQR 3–8).

### Radiation Exposure and Comparison of First and Follow-Up Intervention

**Fig. 1:**
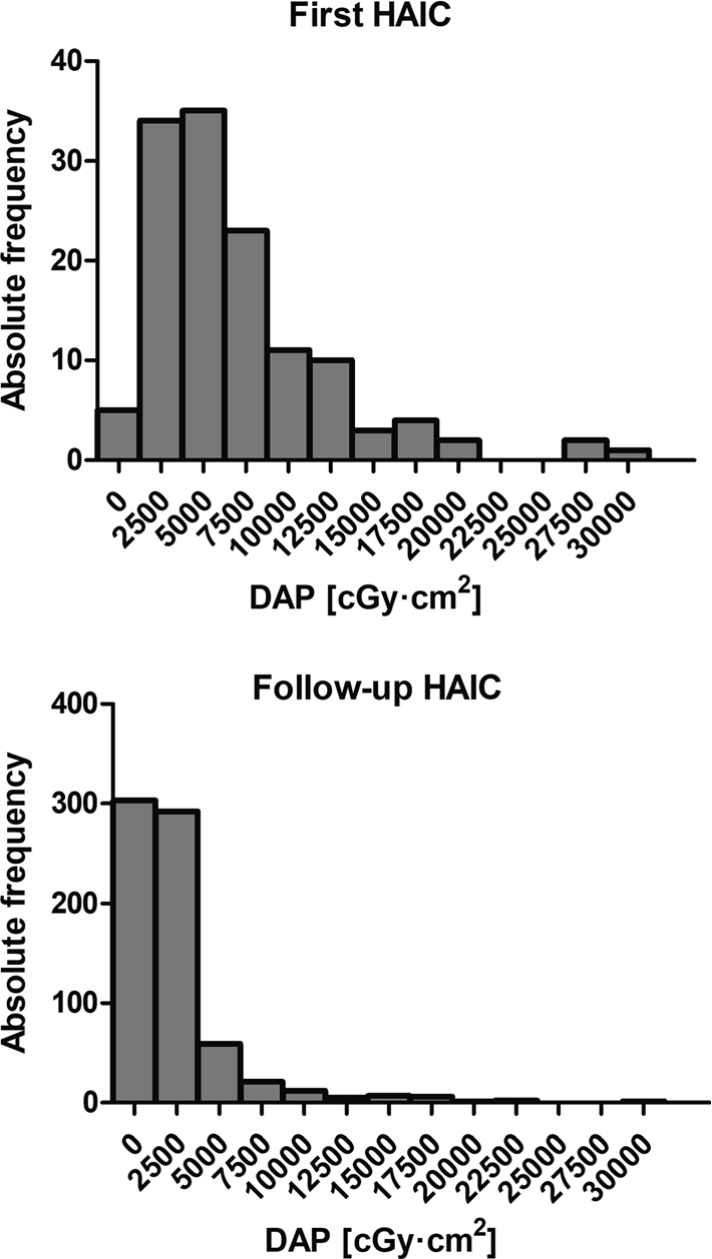
Histograms of the dose area product (DAP) of the first and follow-up hepatic artery infusion chemotherapies (HAIC). The *x*-axis shows the bin centers. In each histogram, one data point above 50,000 cGy·cm^2^ is not depicted in the graph

**Fig. 2:**
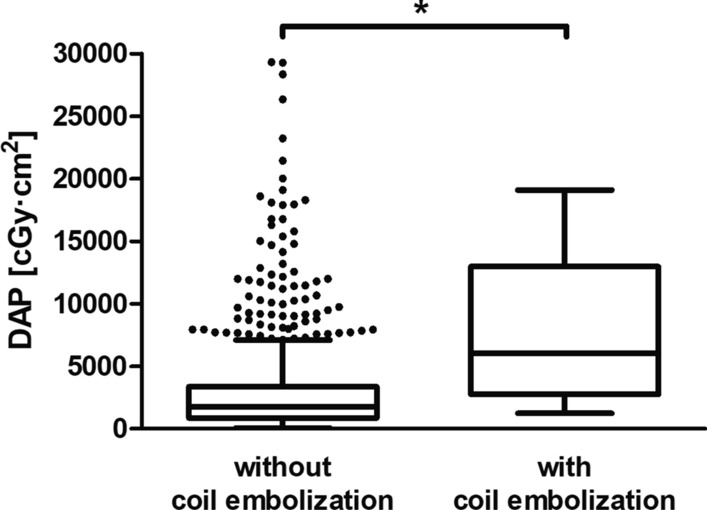
Dose area product (DAP) of hepatic artery infusion chemotherapy (HAIC) with and without coil embolization. Points show outliers outside the Tukey whiskers. Two outliers above 50,000 cGy·cm^2^ are not depicted in the graph for the group without coil embolization. Asterisk indicates statistically significant difference

**Fig. 3:**
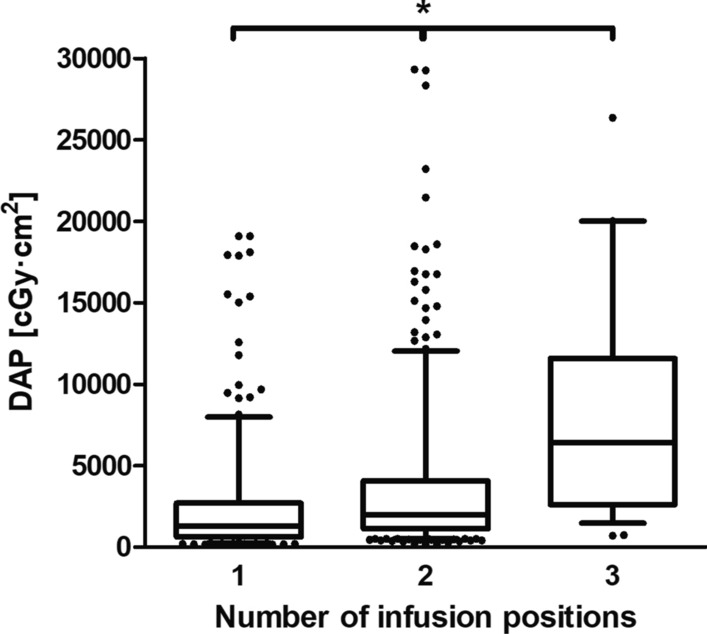
Dose area product (DAP) of hepatic artery infusion chemotherapy (HAIC) as a function of the number of infusion positions. Points show outliers outside the Tukey whiskers. For 1 and 3 infusion positions, one outlier each above 50,000 cGy·cm^2^ is not depicted in the graph. Asterisk indicates statistically significant difference

In the analyzed cohort, median radiation exposure of HAIC in terms of DAP was 1773 cGy·cm^2^ (IQR 884–3688 cGy·cm^2^) (Table 1). Median DAP was significantly higher at first HAIC by a factor of 3.8 (median 5765 cGy·cm^2^, IQR 3160–8804 cGy·cm^2^, 131/841 HAICs) compared with follow-up interventions (median 1502 cGy·cm^2^, IQR 807–2712 cGy·cm^2^, 710/841 HAICs) (*p* < 0.0001) (Fig. 1). In HAICs with coil embolization, the median DAP (6054 cGy·cm^2^, IQR 861–3354 cGy·cm^2^, 32/841 HAICs) was significantly higher by a factor of 3.5 than in interventions without coil embolization (1730 cGy·cm^2^, IQR 2770–12,960 cGy·cm^2^, 809/841 HAICs) (*p* < 0.0001), with 56.3% (18/32) of all coil embolizations performed prior to the first HAIC (Fig. 2). Radiation exposure of HAIC was significantly increased by the number of infusion positions (*p* < 0.0001) (median, IQR): one position 1301 cGy·cm^2^ (633–2717 cGy·cm^2^, 358/841 HAICs), two positions 1985 cGy·cm^2^ (1118–4074 cGy·cm^2^, 454/841 HAICs), three positions 6407 cGy·cm^2^ (2616–11,590 cGy·cm^2^, 29/841 HAICs) (Fig. 3). Thus, median DAP was increased by approximately 53% for 2 positions and by approximately 493% for 3 positions compared with HAIC with only 1 infusion position.

**Table 1:** Radiation exposure in terms of dose area product (DAP) of hepatic artery infusion chemotherapy (HAIC)

HAIC type	*n*	DAP [cGy·cm^2^]		
		25th percentile	Median	75th percentile
Total	841	884	1773	3688
First intervention	131	3160	5765	8804
Follow-up intervention	710	807	1502	2712
with coil embolization	32	2770	6054	12,960
without coil embolization	809	861	1730	3354
1 position	358	633	1301	2717
2 positions	454	1118	1985	4074
3 positions	29	2616	6407	11,590

## Discussion

HAIC is an important treatment option for patients with liver metastases from uveal melanoma. Here, further standardization might lead to a further reduction of radiation exposure for patients and interventional radiologists alike. The results of our study can be subsumed in two key points. First, radiation exposure is significantly increased for the initial intervention compared with follow-up interventions. Second, interventions with coil embolization and interventions with multiple infusion positions are associated with a higher radiation exposure.

For the palliative treatment of liver metastases in UM patients, HAIC is considered a valuable treatment option [1, 11]. As regular repetitions of this intervention are a necessity, dose optimization deserves special consideration and might be beneficial for the patient, the interventional radiologist, and his team alike [12–15].

Our study showed that radiation exposure was significantly higher in the first HAICs than in follow-up interventions, as complete visualization of the liver vasculature was performed with multiple contrast injector DSA series of the arteries supplying the liver. Furthermore, especially additional coil embolization during HAIC resulted in significantly higher radiation exposure. As most coil embolizations were performed during the first HAIC, the disproportionate share of coil embolizations might also contribute to their higher radiation exposure.

Depending on the individual vascular anatomy, it might be necessary to perform HAIC not only in one but in multiple liver arteries to achieve an equal distribution of the chemotherapeutic agent in both liver lobes. However, an increase in infusion positions results in repeated changes of the catheter positions during HAIC. Consecutively, additional radiation exposure is necessary. Here, a second infusion position resulted approximately in a 53% increase in radiation exposure, whereas a third infusion position increased radiation exposure to approximately five times compared to HAICs with one infusion position.

The limitations of our study are its retrospective and single-center study design. However, this preliminary data might serve as an important guide to improve radiation exposure during HAIC.

In conclusion, in uveal melanoma patients with liver metastases, radiation exposure is significantly increased for the first intervention compared with follow-up interventions, for interventions with coil embolization, and for interventions with multiple infusion positions.
